# Microstructural, Mechanical and Corrosion Performance of NbZr1-Ti64 Multi-Material Structure Fabricated via Wire Arc Directed Deposition

**DOI:** 10.3390/ma19183959

**Published:** 2026-09-17

**Authors:** Sainand Jadhav, Duck Bong Kim, Aaron Adams, Sambhaji Kusekar, Tushar Borkar, Showmik Ahsan, Daniel Young

**Affiliations:** 1 Department of Engineering Technology, Kennesaw State University, Marietta, GA 30060, USA; 2 School of Environmental, Civil, Agricultural, and Mechanical Engineering, University of Georgia, Athens, GA 30602, USA; 3 Department of Mechanical and Industrial Engineering, University of Wisconsin, Platteville, WI 53818, USA; 4 Department of Mechanical Engineering, Cleveland State University, Cleveland, OH 44115, USA; 5 Department of Mechanical and Materials Engineering, Wright State University, Dayton, OH 45435, USA

**Keywords:** multi-material structure, NbZr1, Ti64, wire arc directed deposition, corrosion, bimetallic structure, functionally graded materials

## Abstract

This study investigates the fabricability, microstructures, and mechanical and corrosion behavior of a multi-material structure (MMS) composed of niobium alloy (NbZr1) and titanium alloy (Ti64) using a wire arc directed energy deposition process. The microstructure of NbZr1 alloy primarily consisted of equiaxed grains oriented in the rolling direction, while the deposited Ti64 microstructure exhibited ‘banding’ morphology and a basket-weave structure composed of α phase lamellae in a β matrix. The MMS interface revealed good metallurgical bonding and was free from defects such as cracks, pores and intermetallic phases. Niobium diffusion from NbZr1 into the Ti64 alloy resulted in the formation (β-Ti + Nb) of a solid solution which imparted strength to the MMS. Hardness testing showed that microhardness values follow the following trend: NbZr1 substrate > MMS interface > Ti64 deposit. The NbZr1–Ti64 multi-material structure developed in this study exhibited a balanced combination of ductility (22.73% elongation) and moderate tensile strength (254.18 MPa), outperforming most reported NbZr1-Ti64 MMS studies. All tensile specimens failed in a ductile manner on the NbZr1 side. The MMS demonstrated superior corrosion resistance, exhibiting the lowest corrosion current density and corrosion rate compared to its individual counterparts.

## 1. Introduction

Multi-material structures (MMSs) refer to components or assemblies composed of two or more distinct materials, integrated to leverage their individual and combined properties [1]. Multi-material structures offer potentially better performance and numerous advantages over the structures fabricated using conventional single materials. Multi-material structures also encompass terminologies such as bimetals, bimetallic structures (BSs) and functionally graded materials (FGMs) [2]. Functionally graded materials are designed with a smooth transition between two dissimilar materials, whereas bimetallic structures have a sharp transition. Both are engineering approaches used to combine the properties of two different materials to achieve a combination of mechanical, thermal, or chemical properties that a single material cannot provide [3]. The applications of multi-material structures are widespread in sectors such as aerospace, automotive, medical, nuclear, chemical, and energy systems, where multifunctional performance is critical [3,4,5,6].

One of the most widely used alloys in aerospace, biomedical, and energy sectors is Ti-6Al-4V (commonly referred to as Ti64) [7]. Ti64 is a dual-phase (α+β) titanium alloy known for its high strength-to-weight ratio, excellent corrosion resistance, and good biocompatibility [8]. The alloy is extensively used in aerospace structural components, turbine blades, and orthopedic implants due to its capability to withstand high mechanical loads while remaining lightweight. However, Ti64 exhibits limited performance at elevated temperatures (>400 °C) due to a significant reduction in mechanical properties, limiting its applicability in high-temperature environments [9]. On the other hand, niobium-based alloy NbZr1 (niobium with 1 wt.% zirconium) is recognized for its superior high-temperature performance, excellent corrosion resistance in aggressive environments, and outstanding biocompatibility [10]. NbZr1 maintains structural stability and mechanical integrity at temperatures exceeding 1000 °C, making it an ideal candidate for applications in nuclear fuel cladding, chemical reactors, structural components, and aerospace thermal shielding systems [11]. Additionally, zirconium enhances the alloy’s oxidation resistance and solid solution strengthening to the Nb matrix, increasing high-temperature strength while maintaining ductility [10,12].

Integrating NbZr1 with Ti64 in a bimetallic configuration offers a promising pathway to developing advanced structures that leverage the lightweight and high-strength characteristics of Ti64 with the high-temperature and corrosion resistance capabilities of NbZr1. Such hybrid structures can address the critical demands of next-generation aerospace engines, advanced nuclear reactors where structural integrity across a broad temperature range is essential. However, due to the considerable difference in physical and chemical properties between these two alloys—including melting points, thermal expansion coefficients, and elemental diffusivity—the fabrication of a NbZr1/Ti64 MMS presents significant metallurgical challenges [13]. Achieving a strong, defect-free interface between these materials is critical for the performance and reliability of the resulting multi-material structures.

MMSs can be processed by conventional as well as additive manufacturing methods. The conventional manufacturing methods include casting, forging, powder metallurgy, thermal spray deposition, electro-deposition, welding, brazing, diffusion bonding, and the transient liquid phase (TLP) technique [3,14]. Various attempts were made to join Nb alloys and Ti alloys using conventional methods. Torkamany et al. [13,15] investigated the pulsed Nd:YAG laser welding of pure niobium to Ti–6Al–4V. Optimal parameters yielded full-penetration joints without intermetallic formation. Welds exhibited Ti/Nb-rich zones, matching niobium’s tensile strength. Fractures occurred in niobium, indicating sound weld integrity. Gao et al. [16] examined the effect of heat input on pulsed laser-welded Ti6Al4V/Nb joints. Increased heat input improves fusion, reduces defects, and increases the width of the heat-affected zone. Ti- and Nb-rich zones formed without intermetallic formation. Welds exhibited a tensile strength of 250 MPa, with fractures occurring in the Nb base. Zhou et al. [17] successfully laser-welded NiTiNb and Ti6Al4V alloys using a Nb filler. With an increase in laser power, the amount of melted Nb increased, which reduced brittle intermetallics (NiTi, Ti_2_Ni), narrowed IMC regions, and improved joint strength. Franchini et al. [18] also successfully welded a Nb-base alloy (C-103) to Ti6Al4V without cracks using electron beam welding, achieving a tensile strength of 511 MPa. Siqueira [19] et al. studied the microstructure and hardness of fiber laser beam-welded Ti-Nb sheets. The samples were defect-free, and the hardness profile decreased from the top (Ti) to the bottom (Nb). Yulong et al. [20] performed orthogonal parameter optimization for electron beam welding of two dissimilar alloys Nb and Selective Laser Melted TC4. The optimal conditions for attaining the highest tensile strength of 314.1 MPa were an acceleration voltage of 62 kV, welding beam current of 10 mA, and scanning rate of 4.5 mm/s.

Compared with conventional manufacturing methods, additive manufacturing (AM) offers significant advantages in fabricating complex geometries while providing unprecedented flexibility in material composition, structural design, and functional integration, making it particularly attractive for producing multi-material structures [6,21,22,23]. The feasibility of Ti alloy/Nb alloy MMS additive manufacturing has been demonstrated in only a limited number of studies. Jiang et al. [24] fabricated a TC4/Nb multi-material structure using twin wire arc additive manufacturing (WAAM), where the deposited material consisted primarily of α-Ti, β-Ti, and (Nb, Ti) solid solution phases without the formation of brittle intermetallic compounds. Similarly, Jadhav et al. [25] fabricated Ti6Al4V–NbZr1 bimetallic structures using gas tungsten arc welding (GTAW)-based WAAM and reported sound metallurgical bonding with no pores, cracks, or intermetallic compounds at the interface. WAAM offers an attractive route for fabricating multi-material structures with discrete or functionally graded transitions because of its high deposition rate, excellent material utilization, compositional flexibility, and cost-effectiveness for large-scale components [12,13]. Through the appropriate control of process parameters and deposition strategy, WAAM enables the successful integration of dissimilar materials such as NbZr1 and Ti64 while minimizing interfacial defects and tailoring the resulting microstructure.

Despite recent advances, studies on Nb alloy/Ti alloy multi-material structures remain scarce. Although previous studies [24,25] have demonstrated the feasibility of fabricating Ti6Al4V–NbZr1 bimetallic structures using wire arc additive manufacturing (WAAM), a comprehensive understanding of the relationships among interfacial microstructure, mechanical behavior, and corrosion performance remains limited. In particular, the electrochemical corrosion behavior of WAAM-fabricated NbZr1–Ti6Al4V multi-material structures has not been systematically investigated, despite its importance for potential applications in aerospace, biomedical, and chemical processing environments. Therefore, the present study aims to fabricate a NbZr1–Ti6Al4V multi-material structure using WAAM with the deposition of Ti64 on a NbZr1 substrate and systematically investigate its interfacial microstructure, phase constitution, hardness distribution, tensile behavior, fracture characteristics, and electrochemical corrosion response. Particular attention is given to understanding the influence of the dissimilar material interface and associated microstructural evolution on the resulting mechanical and corrosion performance. The findings provide new insights into the process–structure–property relationships governing Nb alloy/Ti alloy multi-material systems and extend existing knowledge beyond fabrication feasibility toward a more comprehensive understanding of their mechanical and corrosion behavior.

## 2. Materials and Methods

### 2.1. Fabrication of Multi-Material Structure and Sample Preparation

**Figure 1:**
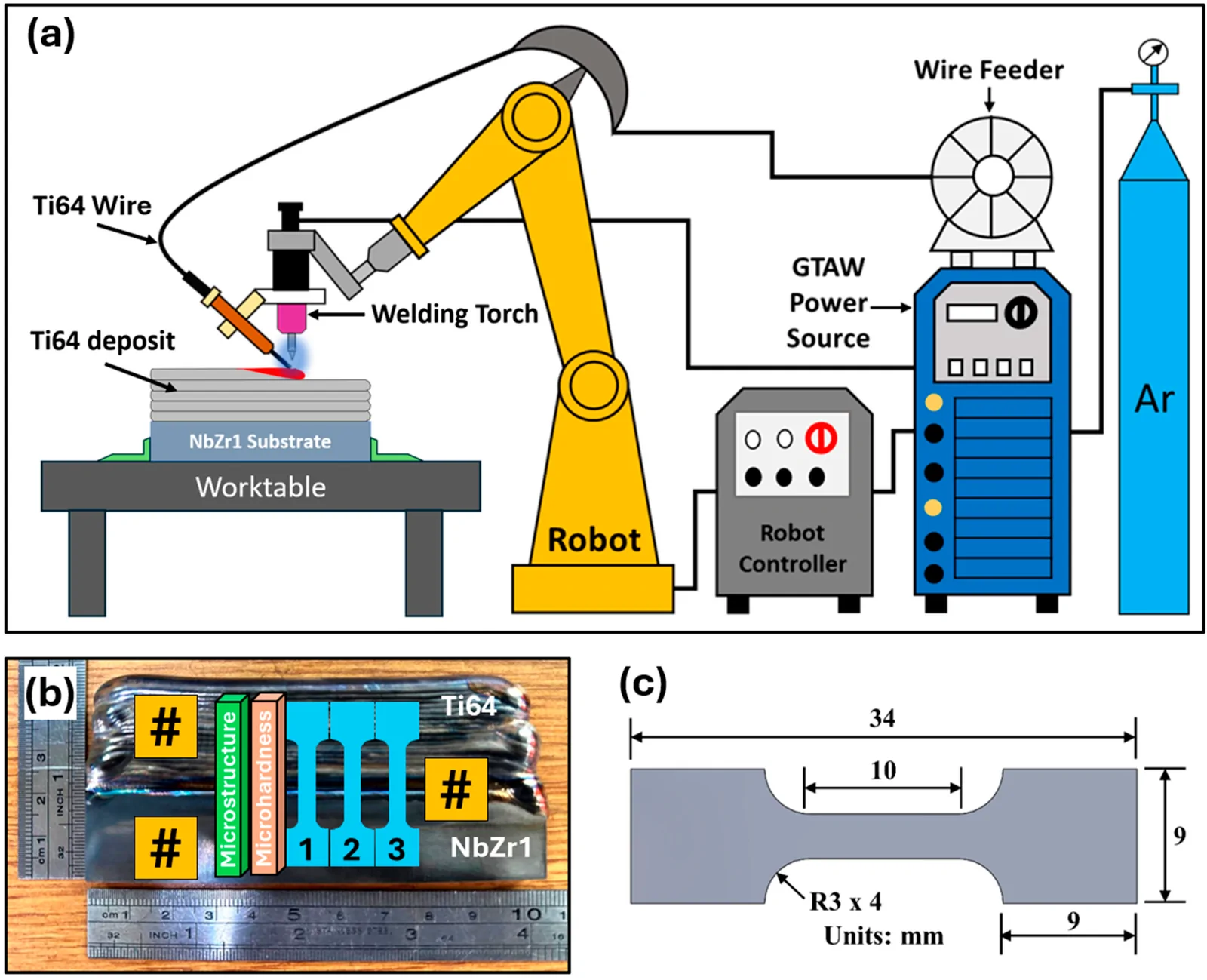
(**a**) GTAW-WAAM setup; (**b**) fabricated NbZr1-Ti64 multi-material wall showing sampling locations for microstructural characterization, microhardness measurements, corrosion# and tensile specimen extraction; (**c**) geometry and dimensions of tensile specimen.

The GTAW-based WAAM system depicted in Figure 1a was used to fabricate a thin-walled, multi-material structure of NbZr1-Ti64. The configuration consists of a 6-axis Fanuc ArcMate 120iC robot with a Fanuc R-J3iB controller, a Miller Dynasty 400 GTA welding power source, and a generic wire feeder. The welding torch was mounted on the face plate of the robot. The robot was programmed by using a teach pendant so that the welding torch follows the programmed path to produce the desired structure. A Ti64 consumable wire, with a diameter of 1.2 mm and specification ERTi-5 (Washington Alloy Company, USA), was used for deposition. Ti64 was deposited on the NbZr1 substrate, measuring 100 mm × 20 mm × 7 mm, provided by ALB Materials Inc. U.S.A. The chemical compositions of the Ti64 consumable wire and NbZr1 substrate are detailed in Table 1. To deposit thin walls, process parameters such as welding current (I), travel speed (TS), and wire feed rate (WFR) were considered. Near-optimal process parameters for depositing multi-layer thin walls were determined based on previous studies [7,26,27], and preliminary experiments were performed to achieve stable arc behavior, uniform bead geometry, and defect-free deposition with adequate fusion while minimizing excessive heat input. High-purity argon (99.999%) served as shielding gas. The same gas served as trailing gas to protect deposition from oxidation and contamination. The process parameters used in this study are shown in Table 2. The rationale for using 200 A current to deposit the first layer of Ti64 was to achieve strong bonding between NbZr1 and Ti64, leveraging the high heat input resulting from the high current. However, starting from the second layer, a current of 140 A was used to achieve lower heat input during the deposition of the subsequent Ti64 layers. This approach helps reduce the residual stress generated in the NbZr1 substrate due to the repetitive thermal cycles experienced during the WAAM process. After the deposition of each layer, the top surface temperature was measured using FLUKE Thermocouple Thermometer (Model: Fluke 51-2 60 HZ) by placing the probe of the thermometer on the surface of the deposited layer, and the subsequent layer was deposited only after the measured surface temperature decreased to approximately 60 °C. A total of 22 layers of Ti64 were deposited. Figure 1b shows the deposited thin-wall structure of NbZr1-Ti64 with a height of 44 mm.

After the deposition process, the thin wall was sectioned along the build direction at specific locations, as shown in Figure 1b, using a metallurgical saw (METCUT-10) to get samples for microstructure characterization and microhardness testing. Samples were hot-mounted and polished. The sample surfaces were then ground with SiC abrasives (grit sizes of 240–1200) and polished using diamond paste solutions of 3 μm and 1 μm in size. The Ti64 side of the polished samples was etched with 3 mL HF + 5 mL HNO_3_ + 100 mL H_2_O, and the NbZr1 side was etched with 10 mL HF + 20 mL HNO_3_ + 10 mL H_2_O. EBSD sample preparation was accomplished by grinding with SiC, followed by polishing down to 1 μm utilizing diamond media, followed by electropolishing as a final step to prepare an EBSD-ready surface. Three tensile specimens, following the ASTM E8 [28] standard, were prepared from the thin wall using wire electrical discharge machining (EDM), with the dimensions illustrated in Figure 1c. For corrosion testing, a square coupon measuring 20 mm × 20 mm × 1.2 mm was sectioned from the NbZr1 substrate, Ti64 deposit, and NbZr1–Ti64 interface regions using wire EDM, as indicated in Figure 1b. The coupons were subsequently ground and polished prior to electrochemical testing.

**Table 1:** Chemical composition (wt.%) of Ti64 consumable wire and NbZr1 substrate [29,30].

	*C*	O	*N*	*H*	*Fe*	*Al*	*V*	*Ti*	*Nb*	*Zr*
*Ti64*	0.05	0.12–0.20	0.03	0.015	0.22	5.5–6.75	3.4–4.5	Bal.	-	-
*NbZr1*	-	-	-	-	-	-	-	-	99	1

**Table 2:** Process parameters.

Process Parameters	Value
**Welding current**	200 A-1st layer, 140A-2nd layer onwards
**Wire feed rate (WFR)**	1800 mm/min
**Travel speed (TS)**	200 mm/min
**Wire diameter**	1.2 mm
**Electrode-to-workpiece distance**	5 mm
**Torch angle**	90°
**Shielding gas composition**	99.999% argon
**Shielding gas flow rate**	15 L/min
**Trailing gas composition**	99.999% argon

### 2.2. Microstructure, Corrosion Characterization and Mechanical Property Testing

The microstructures were analyzed using optical microscope (OM) and scanning electron (SEM) microscope. For optical microscopy, a Nikon SMZ 1500 microscope was used for low-magnification images (up to 10× magnification). A Hitachi SU 7000 field emission scanning electron microscope (SEM) coupled with energy-dispersive spectrometry (EDS) was employed for microstructure and compositional analysis. For EDS analysis, the EDAX Octane Elect Super EDS system with EDAX APEX 2.0 software was used. X-ray diffraction (XRD) was carried out to determine the phases present at the interface using a Rigaku Ultima IV XRD machine. Diffraction patterns were recorded using a D/teX Ultra-High-Speed Detector. Raw data was imported into the PDXL 2.3 software package for phase analysis and comparison with the standard patterns in the ICDD database. Electron beam backscatter diffraction imaging was performed using a JEOL JSM 7900F field emission SEM equipped with an EDAX Hitachi Octane Elect Super EBSD system. EBSD scans were acquired using a step size of 0.06 µm. Data was captured using EDAX TEAM v4.6 and processed using TSL OIM Analysis v8.0 software. Microhardness tests were performed along the build direction of thin walls using a Buehler Wilson VH1202 microhardness tester with a 500 g applied load (Vickers diamond indenter) and dwell time of 10 s. The microhardness tester was equipped with an integrated high-resolution camera and Diamet^TM^ 2.0 software. Mechanical strength was investigated using the computer-controlled, uniaxial tensile testing system (TestResources 810 E4 Electrodynamic Test Machine). Tensile tests were conducted with an elongation rate of 0.001 s^−1^. The fractured surfaces of tensile specimens were examined using the FEI Quanta 200 SEM. Corrosion characterization was performed using a traditional three-electrode electrochemical cell with the test specimen as the working electrode, a platinum mesh as the counter electrode, and a saturated calomel electrode (SCE) as the reference electrode. Potentio-dynamic polarization tests were performed using a BioLogic SP-200 potentiostat. The ground, polished square coupons were washed with ethanol, rinsed with deionized water, and dried before testing. Only a predetermined 10 mm^2^ of the specimen’s surface was exposed to the electrolyte during testing; the rest of the specimen’s surface was kept separate from the testing environment. After stabilizing the open-circuit potential (OCP) for half an hour, measurements were carried out in a 3.5% wt. NaCl solution at room temperature at a scan rate of 1 mV/s. Potentio-dynamic polarization measurements were conducted within the potential range of −0.350 to +0.550 V. Each material underwent a single measurement for the corrosion tests, which were not repeated. Tafel extrapolation was used to calculate corrosion potential and corrosion current density.

## 3. Results and Discussion

### 3.1. Microstructural Analysis

**Figure 2:**
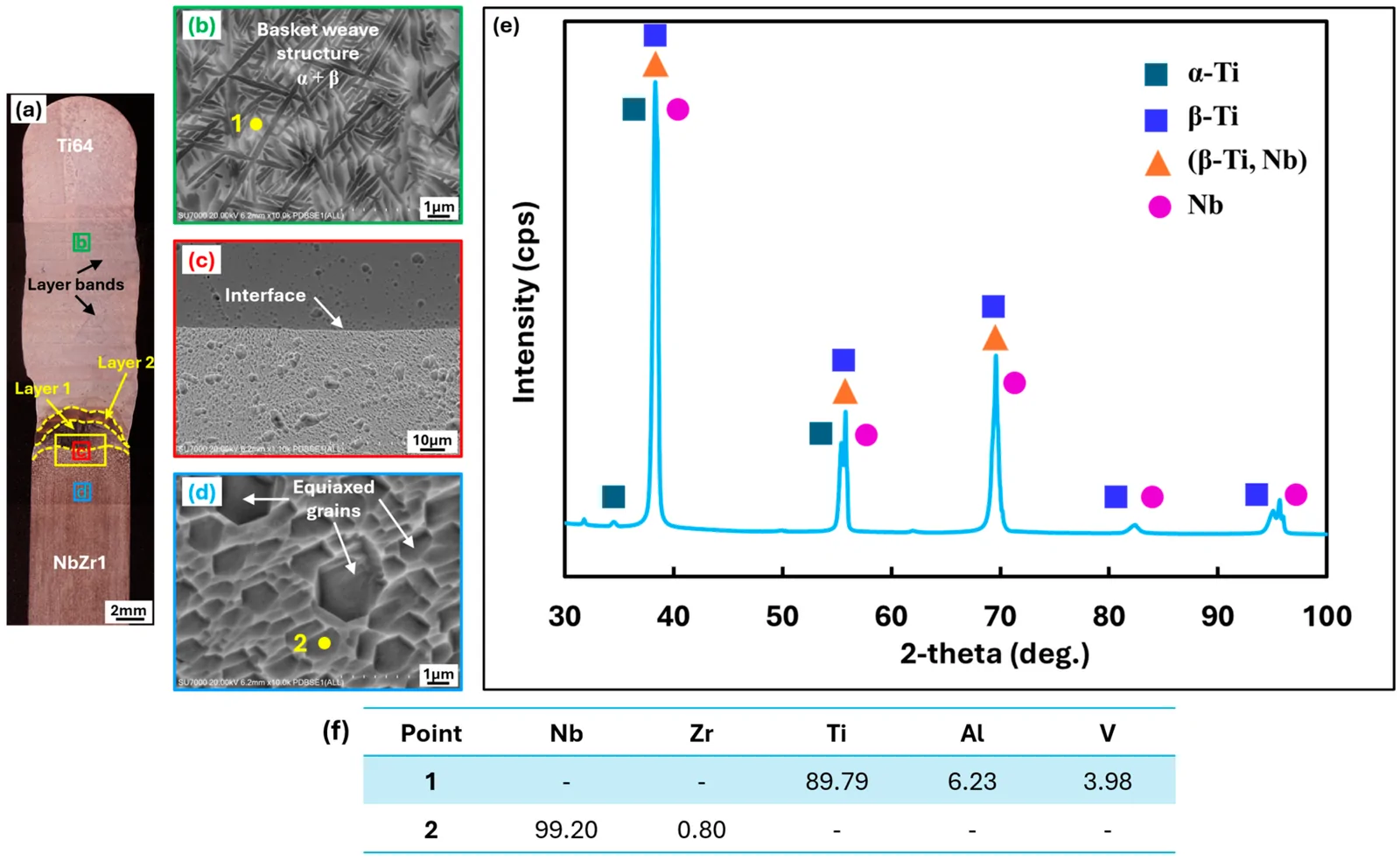
(**a**) Optical microscope image of MMS and SEM images: (**b**) Ti64 deposit; (**c**) MMS interface; (**d**) NbZr1 substrate. (**e**) XRD spectra of MMS interface; (**f**) EDS point scan results of selected points 1 and 2.

Figure 2a shows a cross-sectional optical micrograph of a fabricated NbZr1–Ti64 MMS wall. The deposited structure exhibited a sound build morphology with well-defined layer bands corresponding to successive deposition passes. Two distinct deposited layers were observed above the NbZr1 substrate, indicating stable material deposition and consistent layer formation. As shown in Figure 2a, for the first two layers of Ti64, the layer interfaces can be seen clearly. At the center, the height of the first layer is larger in comparison to the second and subsequent layers. During the deposition of the first layer of Ti64, the NbZr1 substrate also melted partially, which adds to molten Ti64, thus increasing the total volume of molten metal and results in the increased height of the layer at the center. For subsequent layers (second and third layers), only the Ti64 wire was likely melted, resulting in lower subsequent layer heights. After the third layer, parallel bands were observed. Similar ‘banding’ morphology has been observed in many studies on the WAAM of Ti64 because of thermal cycling that causes recrystallization in the α+β region, promoting the nucleation and growth of a secondary α phase and forming a coarsened lamellar structure when cycling conditions are adequate [7,26,27,31,32,33]. Within the top layers of the Ti64 deposited, no parallel bands were observed. A SEM image of the deposited Ti64 region is shown in Figure 2b. A typical basket-weave (Widmanstätten) microstructure consisting of α and β phases was observed throughout the deposited Ti64 layer. The formation of the α+β basket-weave structure is commonly associated with improved strength and toughness in Ti64 alloys due to the interlocking arrangement of α laths within the retained β matrix [7,26,31].

Detailed microstructural and compositional characterization was concentrated within approximately 0.8–1.0 mm across the NbZr1–Ti64 interface because the objective was to capture the substrate, fusion/interface region, and first layer of deposited Ti64, where the strongest effects of substrate dilution, elemental interdiffusion, and rapid compositional transition are expected. The upper Ti64 layers are progressively less influenced by direct dilution from the NbZr1 substrate and were therefore outside the primary scope of the present interface-focused analysis. A higher-magnification SEM image of the NbZr1–Ti64 interface is presented in Figure 2c. A continuous and defect-free metallurgical bond was observed between the deposited Ti64 and the NbZr1 substrate. The interface appeared free from cracks, pores, and interfacial separation, suggesting successful joining between the dissimilar materials. The microstructure of the NbZr1 substrate is shown in Figure 2d. The substrate exhibits equiaxed grains of varying sizes, similar to that observed in the NbZr1 alloy fabricated via powder metallurgy [34]. Furthermore, because the NbZr1 substrate was cold-rolled, the grains are oriented in the rolling direction. To further correlate the observed microstructures of the NbZr1 substrate and Ti64 deposit with their local chemical compositions, EDS point analyses were performed at the selected locations marked as points 1 and 2 in Figure 2b and Figure 2d, respectively, and the corresponding compositions are summarized in Figure 2f. Point 1, located within the Ti64 deposit, shows a Ti-rich composition, with Ti accounting for approximately 89.79 wt.%, together with 6.23 wt.% Al and 3.98 wt.% V, consistent with the Ti64 composition. Point 2 corresponds to the NbZr1 substrate region and exhibits the Nb-rich composition characteristic of the substrate. These EDS results confirm that the distinctly different microstructures observed in the Ti64 deposit and NbZr1 substrate are associated with their respective local chemical compositions.

Figure 2e presents the X-ray diffraction pattern obtained from the NbZr1–Ti64 interfacial region over a 2θ scan range of 30–100°. The analyzed region is indicated by a yellow rectangle in Figure 2a. A 5 mm wide X-ray slit was used to focus the incident X-rays on the interface of the ground and polished samples. Major peaks are observed at 2θ ∼ 35°, 38°, 55°, 69°, 83°, and 95°. Based on the crystal phase analysis of peaks, the interface primarily consists of Nb, α-Ti, β-Ti, and (β-Ti + Nb) phases, and no other intermetallic compounds were observed. The peaks of only one type of solid solution, i.e., (β-Ti + Nb) solid solution, were observed. This is consistent with the Ti-Nb phase diagram, which exhibits strong Ti and Nb solubility. Since the temperature during the deposition of the first layer of Ti64 is higher than the β transition temperature of Ti64, the β-Ti phase predominates in the molten pool, allowing for the transformation of α-Ti alloy into β-Ti. β-Ti and Nb form a (β -Ti + Nb) solid solution in the welding process [20]. According to the binary phase diagrams of Ti-Nb and Nb-V [35], no intermetallic compounds are likely to form in a Ti/Nb and Nb/V couple. Additionally, in the ternary phase diagram of Ti-Nb-Al [36], below 7.5 wt% Al, the formation of intermetallic compounds between niobium and aluminum is not likely. The peaks for the (β-Ti + Nb) solid solution phase are observed at 2θ ∼ 38°, 55°, and 69°, which is consistent with the XRD results reported for electron beam-welded Nb-TC4 joints [20], laser-welded NiTiNb and Ti6Al4V alloys [17], and the multi-WAAM of TC4/Nb heterogeneous alloy [24].

**Figure 3:**
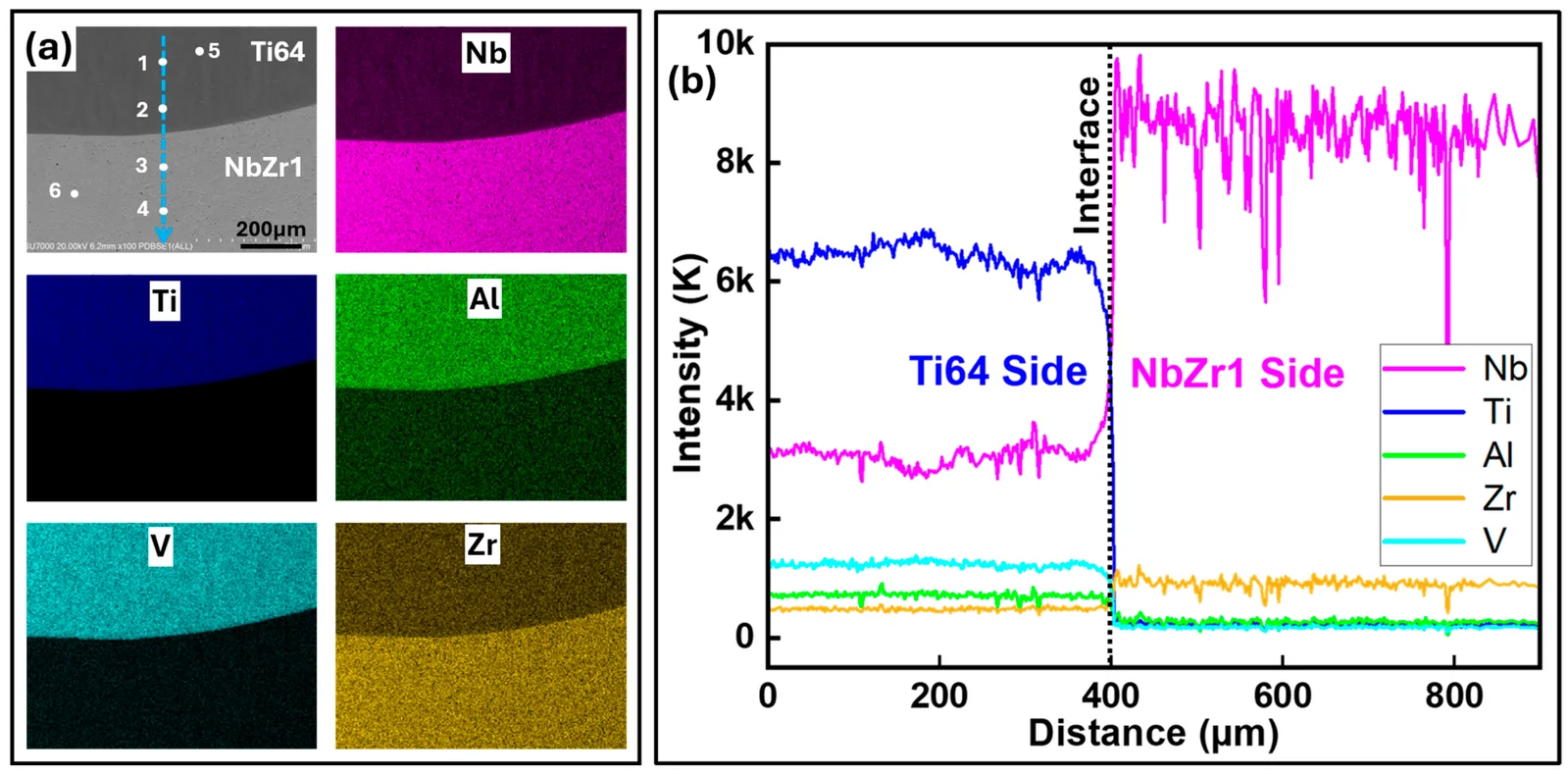
(**a**) EDS area map. (**b**) Line scan results.

Figure 3a presents the EDS elemental area mapping data from the NbZr1–Ti64 multi-material structure, while Figure 3b shows the corresponding line scan results across the interface. A clear compositional transition is observed across the interface, indicating successful bonding between the two dissimilar materials. The elemental area maps show that Ti, Al, and V are predominantly concentrated within the deposited Ti64, whereas Nb and Zr are primarily distributed on the NbZr1 side. Elemental area mapping clearly indicates the diffusion of Nb into the Ti64 deposit, which is corroborated by the EDS point scan analysis. The line scan results further confirm the compositional variation. As the scan traverses from the Ti64 side to the NbZr1 side, the concentrations of Ti, Al, and V decrease sharply, while the Nb concentration increases significantly. The Zr concentration remains relatively low but becomes more pronounced within the NbZr1 region, consistent with the nominal composition of the NbZr1 alloy. The EDS point scan results summarized in Table 3 support the line scan observations. Points 1, 2 and 5, located in the first layer of Ti64 just above the interface, contained approximately 57.85–59.69 wt.% Ti, 28.02–30.26 wt.% Nb, 4.30–4.44 wt.% Al, and 5.41–5.79 wt.% V, indicating the diffusion of Nb from the substrate into molten Ti64 during the deposition of the first layer of Ti64. This composition corresponds to a Ti + Nb solid solution phase [35]. In contrast, points 3, 4, and 6, located within the NbZr1 region, exhibited approximately 96 wt.% Nb with only trace amounts of Ti, Al, and V. However, the Zr concentrations measured locally by SEM-EDS at these points (2.06–3.13 wt.%) were higher than the nominal Zr content (~1 wt.%) of the NbZr1 substrate. This difference should be interpreted considering that Table 1 represents the nominal/bulk alloy composition, whereas EDS provides a localized, semi-quantitative compositional measurement. Local compositional heterogeneity and the uncertainty associated with quantifying a relatively low-concentration element by EDS may contribute to the observed variation. Therefore, the measured local Zr values do not necessarily indicate a corresponding increase in the bulk Zr concentration of the NbZr1 alloy. The presence of small quantities of Ti within the NbZr1 side and Nb within the Ti64 side suggests localized elemental interdiffusion during deposition. The gradual compositional transition across the interface indicates effective metallurgical bonding and strong chemical compatibility between the two alloys. No abrupt compositional discontinuity or enrichment in individual elements was observed, suggesting that significant segregation or formation of brittle intermetallic phases did not occur during processing. The observed elemental interdiffusion is expected to promote interfacial integrity.

**Figure 4:**
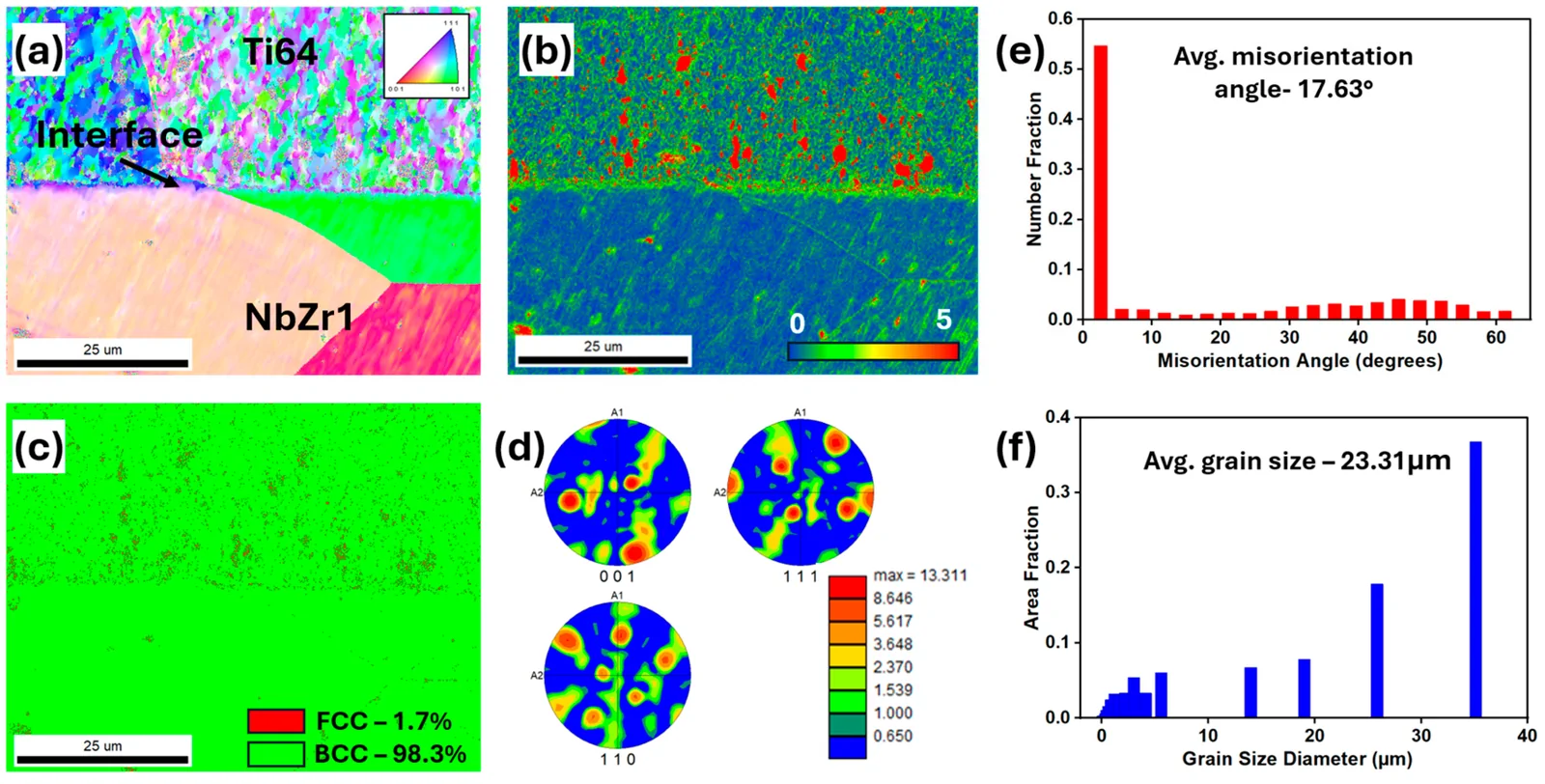
(**a**) EBSD map. (**b**) KAM map. (**c**) Phase fraction. (**d**) Pole figure. (**e**) Misorientation angle. (**f**) Grain size distribution at interface of NbZr1-Ti64 multi-material structure.

A representative EBSD analysis of the NbZr1–Ti64 interfacial region is shown in Figure 4. Figure 4a presents the inverse pole figure (IPF) map across the interface. The NbZr1 substrate exhibits relatively coarse grains, whereas the WAAM-deposited Ti64 region shows a considerably finer microstructural morphology. This difference can be attributed to the distinct solidification and thermal histories experienced by the substrate and deposited material during the WAAM process. The fine basket-weave morphology observed in the Ti64 deposit by SEM is not clearly resolved in the EBSD map at the selected scan scale. The Kernel Average Misorientation (KAM) map in Figure 4b reveals spatial variations in local crystallographic misorientation across the NbZr1–Ti64 interfacial region. Relatively low KAM values are observed within much of the NbZr1 substrate, whereas comparatively higher KAM values occur within portions of the WAAM-deposited Ti64 and in the vicinity of the interface. Higher KAM values indicate increased local lattice curvature and crystallographic misorientation and are commonly associated with greater local strain accumulation and geometrically necessary dislocation density, whereas low KAM values indicate a more uniform crystal orientation and comparatively lower stored deformation. The increased local misorientation near the interface may result from the heterogeneous thermal cycles, differences in thermophysical properties between NbZr1 and Ti64, and the associated deformation generated during deposition and cooling. However, KAM analysis does not provide a direct quantitative measurement of residual stress; therefore, the observed KAM variations are interpreted here in terms of local crystallographic misorientation and strain accumulation rather than residual stress.

The phase map in Figure 4c shows that approximately 98.3% of the analyzed area was indexed as BCC, while approximately 1.7% was indexed as FCC. Importantly, both Nb and β-Ti possess BCC crystal structures; consequently, conventional EBSD phase indexing based on crystallographic symmetry alone cannot unambiguously distinguish BCC Nb from BCC β-Ti. Therefore, the dominant BCC fraction observed in Figure 4c should not be considered direct evidence of Nb diffusion into the Ti64 deposit. Instead, the EBSD phase map provides crystallographic information that should be considered together with the EDS compositional analysis. The EDS results confirm the presence of Nb within the Ti-rich interfacial/deposited region, providing complementary evidence of Nb diffusion. Moreover, because Nb is a β-stabilizing element in Ti alloys, its incorporation into the Ti64 region may promote the stabilization of the BCC β-Ti phase. The pole figures shown in Figure 4d indicate preferred crystallographic orientations within the analyzed region. However, the interpretation of the texture should be approached cautiously because the analyzed area contains a relatively small number of large NbZr1 grains together with the finer Ti64 microstructure, which can influence the apparent orientation intensity. Figure 4e shows the grain boundary misorientation distribution, with an average misorientation angle of 17.63°. A pronounced fraction of low-angle misorientations is observed, indicating the presence of local lattice rotations and subgrain structures that may have developed due to the heterogeneous thermal and deformation history associated with the WAAM process. A weaker population of misorientations is also observed in the approximately 40–50° range. Figure 4f presents the grain size distribution, yielding an overall average grain size of 23.31 μm for the analyzed region. However, because the NbZr1 substrate and Ti64 deposit exhibit substantially different grain morphologies and characteristic length scales, this combined average should be interpreted cautiously, and separate analyses would be required for a rigorous quantitative comparison of their respective grain sizes. The combined OM, SEM, and XRD analyses demonstrate that the NbZr1–Ti64 multi-material structure possesses a defect-free metallurgical interface, a characteristic α+β basket-weave microstructure in the deposited Ti64 region, and a stable equiaxed grain structure in the NbZr1 substrate. The absence of interfacial defects and brittle intermetallic compounds is expected to contribute positively to the mechanical integrity and corrosion resistance of the fabricated MMS.

### 3.2. Mechanical Properties

#### 3.2.1. Microhardness

**Figure 5:**
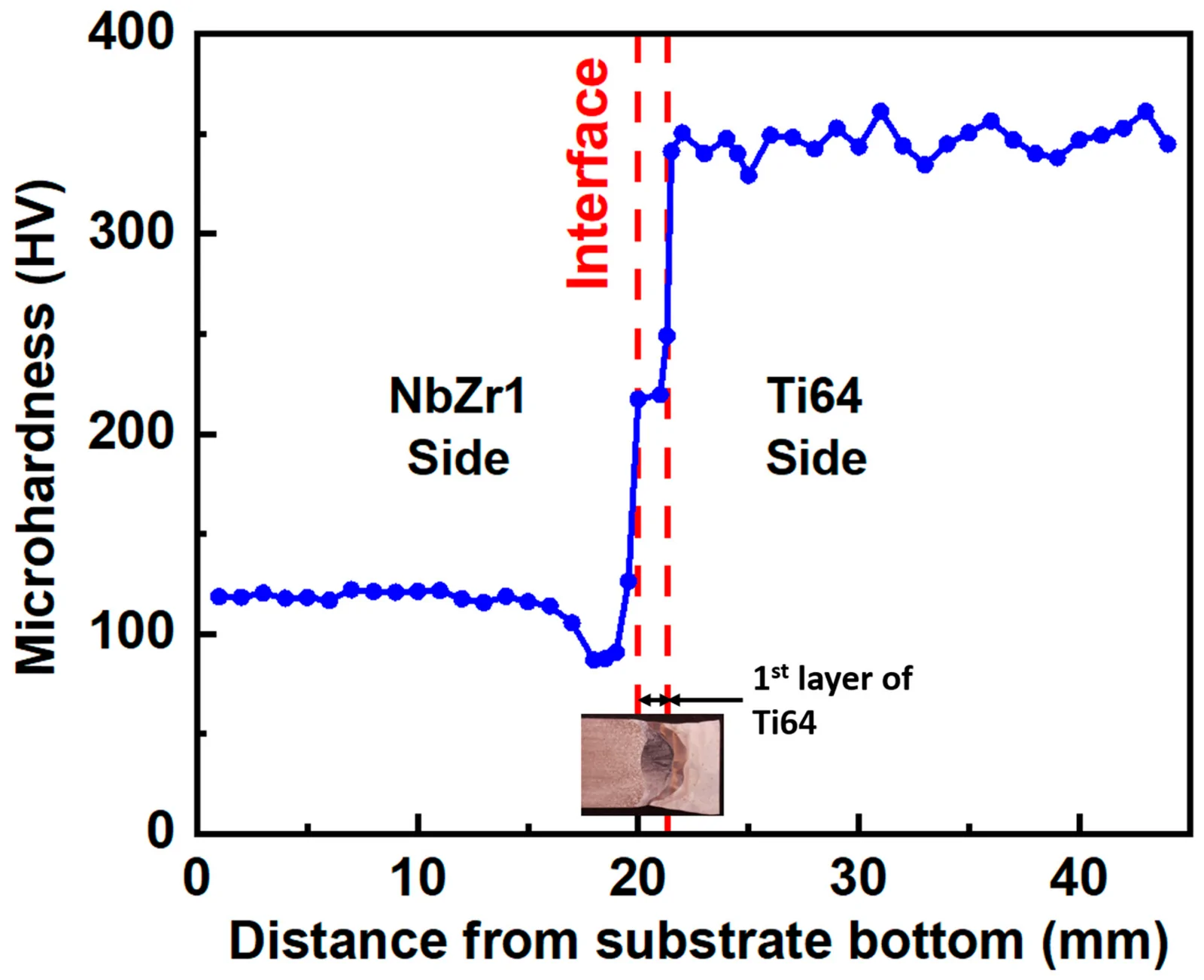
Hardness profile of NbZr1-Ti64 MMS.

In order to study the microhardness distribution of the NbZr1-Ti64 MMS prepared by WAAM, microhardness measurements along the building direction were carried out, as shown in Figure 5. The microhardness values follow the following trend: NbZr1 substrate > MMS interface > Ti64 deposit. The microhardness of the NbZr1 substrate varied between 105 and 122 HV, with an average value of 117 HV, which is comparable to conventionally fabricated NbZr1 (121.5± 2.4 HV) [12,30]. On the NbZr1 side just below the bimetallic interface (heat-affected zone of NbZr1), a reduction in hardness (87 HV) was observed. This reduction in hardness is due to the coarsening of grains in the heat-affected zone of NbZr1. Similar phenomena have been reported in the electron beam welding of Nb sheets [37,38]. At the interface, microhardness slightly increased to 217 HV. Within the first layer of the deposit, hardness further increased to 249 HV. The increase in hardness at the interface and first layer can be attributed to the solid solution strengthening provided by Ti and Nb. From the second layer onwards (Ti64 deposit), the hardness ranges from 329 to 361 HV, with an average value of 346 HV. Similar hardness values have been observed in the WAAM of Ti64 [7,26,27,32,33].

#### 3.2.2. Tensile Properties

**Figure 6:**
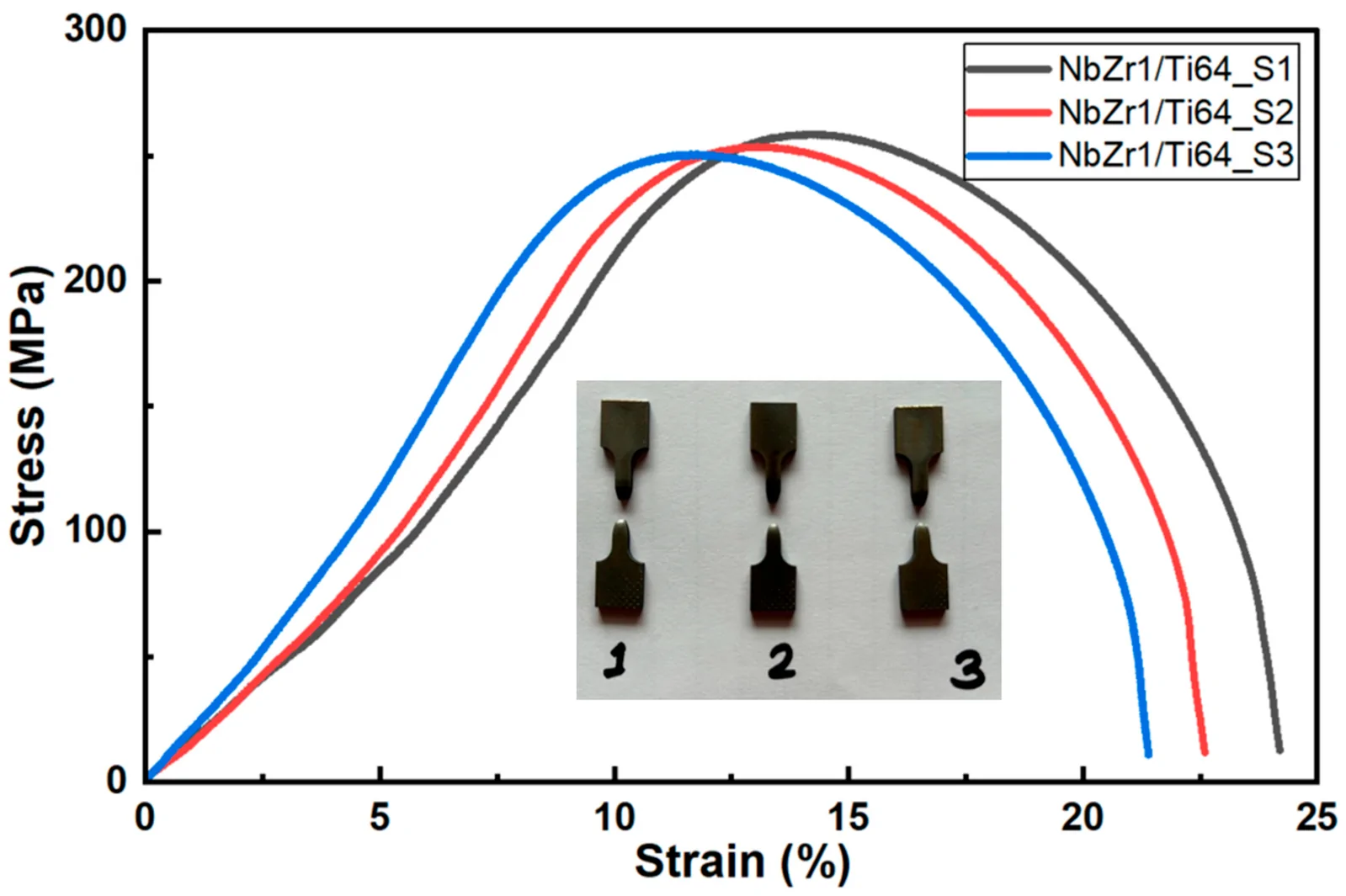
Stress–strain curves of NbZr1-Ti64 MMS.

The engineering stress–strain curves of the NbZr1–Ti64 MMS tensile specimens are shown in Figure 6. All three specimens exhibited similar deformation behavior, indicating the good repeatability of the fabrication process. The specimens showed an initial linear elastic region followed by substantial plastic deformation prior to fracture, suggesting a predominantly ductile failure mode. The average ultimate tensile strength (UTS) and elongation were measured as 254.18 ± 3.37 MPa and 22.73 ± 1.15%, respectively. The small standard deviation in both strength and elongation demonstrates the consistency of the deposited structure and the reliability of the metallurgical bond formed between NbZr1 and Ti64. The tensile specimens sustained significant plastic deformation after yielding, as evidenced by the extended strain-hardening region and high elongation values observed in the stress–strain curves. The fractured specimens shown in the inset of Figure 6 further confirm substantial necking on the NbZr1 side before failure, which is characteristic of ductile deformation. Following tensile testing, the fractured specimens were examined to identify the failure location. All specimens fractured within the NbZr1 substrate region, away from the NbZr1–Ti64 interface. No fracture was observed at or near the interface, indicating that the metallurgical bond between NbZr1 and Ti64 was sufficiently strong to sustain the applied tensile loading. Therefore, although the NbZr1–Ti64 multi-material structure exhibited excellent ductility, its moderate ultimate tensile strength was primarily governed by the comparatively lower strength of the NbZr1 substrate rather than by interfacial failure.

A comparison of the tensile properties of previously reported Nb/Nb alloy–Ti64 multi-material structures fabricated using various manufacturing techniques is presented in Table 4. Although the NbZr1-Ti64 MMS fabricated in this study has a lower UTS than reported in previous studies [13,24,25], which reported strengths ranging from approximately 269 to 610 MPa, it significantly outperformed in terms of elongation. The reduced strength can be attributed to the relatively lower strength of the NbZr1 substrate and the microstructural heterogeneity associated with the dissimilar material interface. The UTS obtained in the present study (254.18 ± 3.37 MPa) was lower than the 543.5 MPa reported for the Ti64–NbZr1 WAAM structure in [25]. This difference can primarily be attributed to the differences in processing conditions and the deposition sequence. In a previous study [25], NbZr1 was deposited onto a Ti64 substrate, whereas Ti64 was deposited onto a NbZr1 substrate in the present study. Reversing the deposition sequence alters heat flow, dilution, elemental diffusion, solidification behavior, and interfacial thermal cycles, producing different microstructures, phase distributions, and hardness gradients. These differences likely promoted localized deformation within the transition region and contributed to the lower UTS observed in the present study. Nevertheless, the UTS obtained in the present study is comparable to that reported for pulsed laser-welded Ti6Al4V–Nb joints (250 MPa) [16]. Despite the moderate tensile strength, the fabricated NbZr1–Ti64 MMS demonstrated superior ductility compared with most reported Nb/Ti64 dissimilar joints. The measured elongation of 22.73% exceeded the values reported for Ti64–NbZr1 WAAM structures (3.9%) [25], Ti64–Nb WAAM structures (14.59–17.77%) [24], and electron beam-welded Nb–Ti64 joints (5.2–6.6%) [20]. The elongation was also slightly higher than that reported for Nd:YAG laser-welded Ti6Al4V–Nb joints (~20%) [13]. The enhanced ductility indicates that the interface could accommodate strain without premature crack initiation or catastrophic failure, thereby promoting stable plastic deformation during tensile loading. Overall, the tensile results demonstrate that the NbZr1–Ti64 multi-material structure fabricated in this study provides an attractive combination of moderate tensile strength and excellent ductility, making it suitable for applications where deformation tolerance and structural integrity are more critical than the maximum load-bearing capacity. The high elongation achieved further suggests effective metallurgical bonding and the absence of brittle intermetallic compounds at the NbZr1–Ti64 interface.

**Figure 7:**
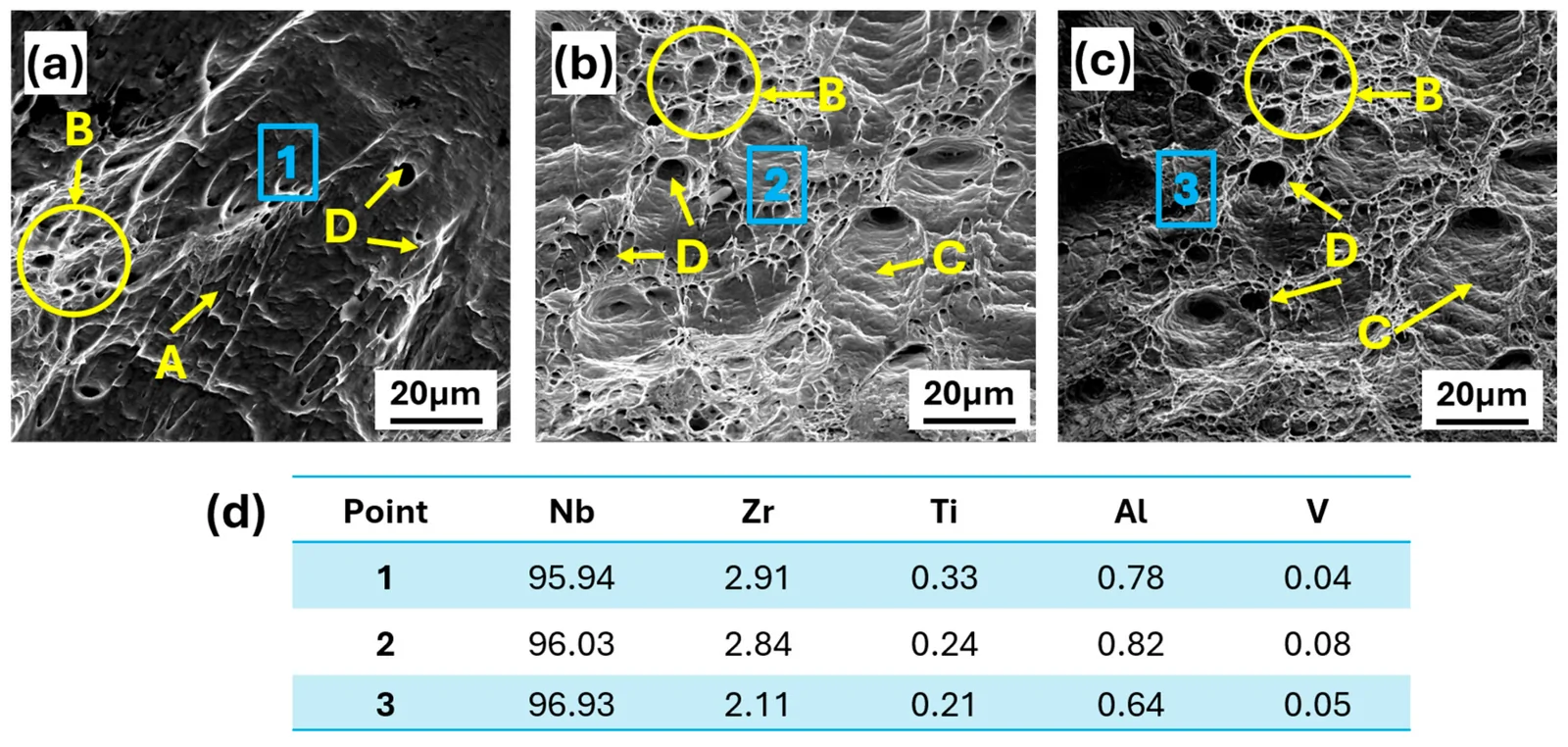
(**a**–**c**): SEM images of fractured tensile specimens [A: Plastic flow region/deformation bands. B: Microvoid coalescence region (fine dimples). C: Tear ridges and fibrous ligaments. D: Elongated dimples]. (**d**): EDS point scan results of fracture surfaces of tensile samples.

In order to systematically reveal the fracture mechanism of tensile specimens, the fracture morphology was analyzed by scanning electron microscopy. The fracture surfaces of the NbZr1–Ti64 multi-material tensile specimens after tensile testing are shown in Figure 7a–c. Fractographic analysis revealed a predominantly ductile fracture mode, consistent with the high elongation of 22.73 ± 1.15% obtained during tensile testing. All specimens exhibited extensive plastic deformation accompanied by microvoid nucleation, growth, and coalescence, indicating significant energy absorption prior to failure. Figure 7a shows plastically deformed regions (A) and clusters of fine microvoids (B), which likely nucleated around microstructural heterogeneities and subsequently coalesced during crack propagation. The elongated cavities (D) represent stretched dimples formed under tensile loading. In Figure 7b, a higher density of equiaxed and elongated dimples is observed. The circled region (B) highlights microvoid coalescence, while feature (C) corresponds to tear ridges and fibrous ligaments formed between neighboring voids during final fracture. The elongated depressions (D) indicate void growth and linkage along the crack path. Figure 7c further confirms the ductile nature of failure, displaying numerous interconnected large and small dimples across the fracture surface. The absence of cleavage facets, river patterns, and intergranular cracking indicates that fracture occurred primarily through a microvoid coalescence mechanism. The extensive dimpled morphology and plastic flow features suggest strong metallurgical bonding at the NbZr1–Ti64 interface, enabling substantial plastic deformation without premature interfacial failure.

To confirm the fracture location, EDS point scan measurements were performed on the fractured surfaces of the tensile specimens at locations 1, 2, and 3 indicated in Figure 7a–c, and the corresponding chemical compositions are presented in Figure 7d. All three locations exhibited Nb as the predominant element, with concentrations of approximately 95.94–96.93 wt.%, together with approximately 2.11–2.91 wt.% Zr. In contrast, only trace amounts of Ti, Al, and V were detected. The measured compositions are therefore characteristic of the NbZr1 substrate rather than the deposited Ti64 or the NbZr1–Ti64 interfacial region. These EDS results confirm that tensile fracture occurred within the NbZr1 substrate.

### 3.3. Corrosion Performance

**Figure 8:**
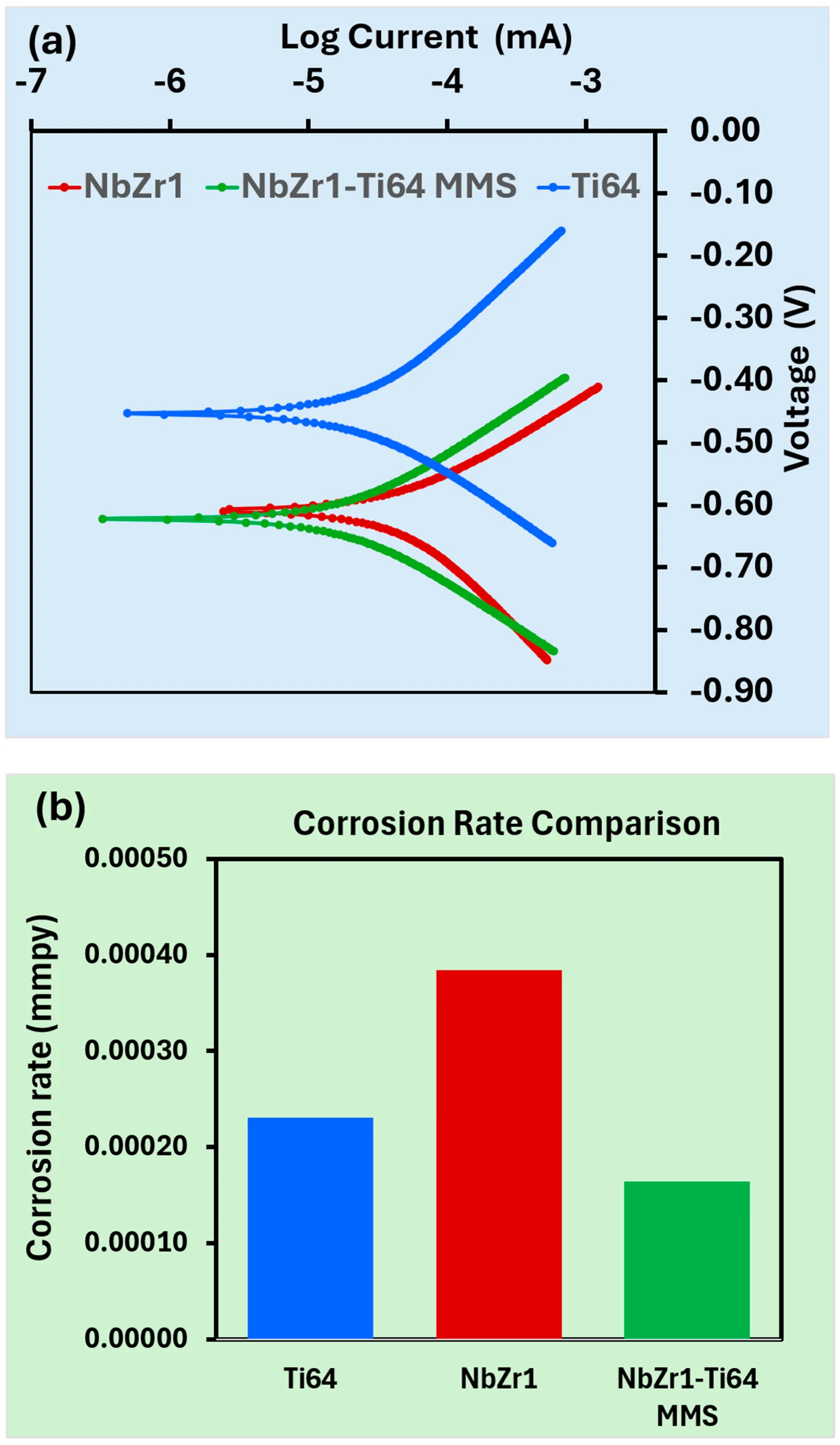
(**a**) Potentio-dynamic polarization (Tafel) curves for NbZr1-Ti64 MMS in 3.5 wt.%NaCl solution. (**b**) Corrosion rate comparison.

The corrosion behavior of the NbZr1–Ti64 multi-material structure was evaluated using potentio-dynamic polarization tests in a 3.5 wt.% NaCl solution, and the resulting Tafel curves are presented in Figure 8a. Table 5 depicts electrochemical corrosion parameters derived from potentio-dynamic polarization tests. The corrosion potential (E_corr_) and corrosion current density (I_corr_) were determined using Tafel extrapolation. Linear regression was performed on the approximately linear portions of the cathodic branches over −0.85 to −0.61 V for NbZr1, −0.84 to −0.63 V for the NbZr1–Ti64 MMS, and −0.66 to −0.46 V for Ti64 and on the anodic branches over −0.61 to −0.41 V, −0.63 to −0.40 V, and −0.46 to −0.16 V, respectively. Data immediately adjacent to E_corr_ and regions deviating from linear Tafel behavior were excluded from the fitting. The intersection of the extrapolated anodic and cathodic Tafel lines was used to determine E_corr_ and I_corr._ The corrosion rate was calculated by potentiostat software (version 11.72) after performing the Tafel fit using the measured values. The corrosion rate (CR) was calculated using the following [39]:

$$CR=3.27\times 10^{-3}\frac{I_{corr}EW}{\rho }$$

where CR is expressed in mm/year and $I_{corr}$ in μA cm^−2^, EW is the equivalent weight in g equivalent^−1^, and ρ  is material density in g cm^−3^. For the NbZr1–Ti64 MMS, the equivalent weight and density used in the calculation were determined based on the composition of the electrochemically exposed surface.

The polarization curves indicate that the materials under investigation exhibit signs of passivation based on their anodic polarization behavior. However, noticeable differences were observed in their electrochemical responses. The NbZr1–Ti64 MMS exhibited the lowest corrosion current density (0.019 μA cm^−2^) among the investigated materials, compared with 0.026 μA cm^−2^ for Ti64 and 0.045 μA cm^−2^ for NbZr1. Since corrosion current density is directly proportional to the corrosion rate, the lower I_corr_ value of the multi-material structure indicates superior corrosion resistance [40,41]. The measured corrosion potential of the NbZr1–Ti64 MMS was −0.6266 V, which was more negative than those of Ti64 (−0.4552 V) and NbZr1 (−0.6086 V). Although the E_corr_ of the NbZr1–Ti64 MMS was more negative (−0.6266 V) than that of Ti64 (−0.4552 V) and NbZr1 (−0.6086 V), this negative shift does not always mean that the corrosion resistance is lower. While I_corr_ is more closely linked to the corrosion rate and kinetics of the corrosion response, E_corr_ indicates the material’s electrochemical potential under test conditions. Similar results have been observed for Ti-based alloys, where corrosion resistance has been assessed by considering passive film properties and E_corr_ in addition to I_corr_ rather than E_corr_ alone [42]. Despite having a slightly negative E_corr_, the NbZr1–Ti64 MMS showed the lowest I_corr_ (0.019 μA cm^−2^) and corrosion rate (0.000164 mmpy) in the current study, showing suppressed corrosion kinetics. The complex electrochemical response of the multi-material structure and the development of protective oxide layers at the Ti64-NbZr1 interface regions may be related to this behavior.

The corrosion rate comparison shown in Figure 8b further confirms the enhanced corrosion resistance of the NbZr1–Ti64 MMS. The multi-material structure exhibited the lowest corrosion rate among the tested materials, whereas NbZr1 showed the highest corrosion rate, and Ti64 displayed intermediate behavior. The improved corrosion resistance may be associated with the formation of stable Ti-, Nb-, and Zr-containing surface oxides [43,44], while the direct surface characterization would be required to confirm their composition and distribution. The I_corr_ values are affected by the passive oxides’ composition, continuity, compactness, and resistance to breakdown caused by chloride in addition to their stability. Although Al_2_O_3_ may contribute to the passive film of Ti64, considerable ZrO_2_ production cannot be assumed for NbZr1 because of its low Zr concentration. Because of the increased local Zr concentration, ZrO_2_ may have a limited contribution to the NbZr1–Ti64 MMS. Passivation is the predominant corrosion behavior and considers the circumstances under which chloride ions may cause the passive coating to become unstable and start localized corrosion. Furthermore, the metallurgical bonding achieved between NbZr1 and Ti64 likely contributed to the development of a compact and continuous passive film across the interface region. The absence of brittle intermetallic compounds and significant interfacial defects, as observed in the microstructural analysis, may have minimized localized galvanic effects and prevented preferential corrosion attack at the dissimilar material interface. Overall, the potentio-dynamic polarization results demonstrate that the fabricated NbZr1–Ti64 multi-material structure possesses superior corrosion resistance compared with the individual Ti64 and NbZr1 materials. The combination of a low corrosion current density, reduced corrosion rate, and stable passive behavior highlights the potential of the NbZr1–Ti64 MMS for applications requiring both mechanical reliability and enhanced corrosion resistance in chloride-containing environments.

## 4. Conclusions

The NbZr1–Ti6Al4V multi-material structure was successfully fabricated using the wire arc directed deposition process, and the relationships between microstructure, mechanical properties, and corrosion behavior were systematically investigated. The major conclusions are summarized as follows:

1. A defect-free metallurgical bond was successfully achieved between the NbZr1 substrate and the wire arc-deposited Ti64 without the formation of cracks, pores, or interfacial delamination at the interface. The interfacial microstructure exhibited a continuous diffusion zone characterized by Nb diffusion into the Ti64 deposit, promoting the formation of a Nb-stabilized (β-Ti + Nb) solid solution while suppressing the formation of brittle intermetallic compounds. EBSD characterization revealed a predominantly BCC microstructure near the interface with an average grain size of approximately 23.3 μm and an average grain misorientation angle of 17.6°. The interface exhibited relatively weak crystallographic texture and a high fraction of low-angle grain boundaries, indicating effective recovery during repeated thermal cycling and contributing to strong interfacial bonding.
2. The hardness profile showed a gradual transition across the interface without abrupt changes, reflecting the diffusion-controlled compositional gradient and absence of brittle intermetallic compounds. The NbZr1–Ti64 multi-material structure exhibited a combination of moderate tensile strength (254.18 ± 3.37 MPa) and excellent ductility (22.73 ± 1.15%), demonstrating superior ductility compared with most previously reported studies. Fractographic analysis confirmed a ductile microvoid coalescence fracture mechanism with extensive plastic deformation and no evidence of brittle interfacial failure.
3. Electrochemical testing in 3.5 wt.% NaCl solution showed that the NbZr1–Ti64 multi-material structure exhibited superior corrosion resistance compared with the individual constituent materials, as evidenced by the lowest corrosion current density and the highest polarization resistance. The enhanced corrosion performance is attributed to the formation of a stable passive oxide layer and the chemically graded diffusion region at the interface.
4. The combined microstructural stability, good mechanical performance, and improved corrosion resistance demonstrate that wire arc directed deposition is an effective manufacturing route for producing NbZr1–Ti64 multi-material structures, offering significant potential for aerospace, biomedical, nuclear, and other high-performance engineering applications requiring corrosion-resistant and high-temperature structural components.

## Figures and Tables

**Table 3:** EDS point scan results of selected points in Figure 3a (wt.%).

Point	Ti	Nb	Al	V	Zr
**1**	58.33	29.69	4.30	5.61	2.07
**2**	57.85	30.26	4.30	5.41	2.18
**3**	0.19	95.92	0.64	0.13	3.13
**4**	0.21	96.08	0.67	0.19	2.85
**5**	59.69	28.02	4.44	5.79	2.06
**6**	0.34	96.04	0.55	0.25	2.82

**Table 4:** Comparison of tensile properties of Nb/Nb alloy–Ti64 multi-material structures.

Nb/Nb Alloy–Ti64 Multi-Material Fabrication Method	UTS (MPa)	EL (%)
NbZr1-Ti64 (this study)	254.18 ± 3.37	22.73 ± 1.15
Ti64–NbZr1 (WAAM) [25]	543.5	3.9
Ti64-Nb (WAAM) [24]	Long. Dir. 452.96 Trans. Dir. 610.33	14.59 17.77
Ti6Al4V–Nb (Nd:YAG laser welding) [13]	269	~20
Ti6Al4V–Nb (pulsed laser welding) [16]	250	-
Nb (conventional) – Ti64 Nb (SLM) – Ti64 (electron beam welding) [20]	314.1 ± 7.8 293.2 ± 0.9	6.6 ± 1.0 5.2 ± 0.7

**Table 5:** Electrochemical corrosion parameters derived from potentio-dynamic polarization tests.

Material	Ecorr (V)	Icorr (µAcm^−2^)	Corrosion Rate (mmpy)
Ti64	−0.4552	0.026	0.000231
NbZr1	−0.6086	0.045	0.000384
NbZr1-Ti64 MMS	−0.6266	0.019	0.000164

## Data Availability

The original data presented in the study are openly available in FigShare at https://doi.org/10.6084/m9.figshare.33194601.
